# Families and Collective Futures: Developing a Program Logic Model for Arts-Based Psychosocial Practice With South African Rural Communities

**DOI:** 10.3389/fpsyg.2021.745809

**Published:** 2021-12-08

**Authors:** Dominik Havsteen-Franklin, Marlize Swanepoel, Jesika Jones, Uné Conradie

**Affiliations:** ^1^Central and North West London NHS Foundation Trust, London, United Kingdom; ^2^Arts and Humanities, Brunel University London, Uxbridge, United Kingdom; ^3^Sp(i)eel Creative Arts Therapies Collective, Cape Town, South Africa

**Keywords:** logic model, psychosocial, arts, community resilience, collective trauma

## Abstract

**Aim:** This aim of this study is to describe the development of a program logic model to guide arts-based psychosocial practice delivered in rural South African farming communities affected by transgenerational traumas.

**Background:** The rationale for developing a program logic model for arts-based psychosocial practice in South Africa was based on the lack of evidence for effective community arts-based psychosocial interventions for collective trauma, unknown consensus about best practices and the need for developing cogent collective psychosocial practices. Further to this, the aims and benefits of the practice required clarity given the psychosocial complexity of the environment within which the practices for this population are being offered. The logic model offers a valuable resource for practitioners, participants and funders to understand the problem being addressed, how practice is defined, as well as the impact of practice and on intermediate and longer term goals.

**Methods:** The authors used a systematic iterative approach to describe the operationalization of arts-based psychosocial practice. This resulted in the design of the logic model being informed by data from focus groups, an overview of the literature regarding transgerenational trauma in this population, operational policies and organizational documents. The development of the logic model involved actively investigating with practitioners their work with remote farming communities. We thematised practitioners practice constructs to identify salient practice elements and their relationship to perceived benefits and lastly feedback from practitioners and participants following implementation to make adjustments to the logic model.

**Results:** The results were clearly identified in the form of visual mapping using the design of a program logic model. The logic model was divided into 5 parts and was verified by practitioners following implementation. The parts of the program logic model are (Part 1) main presenting problem, (Part 2) operational processes, (Part 3) practice elements, (Part 4) benefits, and impact and (Part 5) review.

## Introduction

Recent advances in the international development and remit of arts therapies and psychosocial practice (Fancourt and Finn, 2019) has implications for practice development and the practice infrastructures that support guidance and research for clinicians working in the field. The logic model is a useful tool for structuring programs and planning for environments where clear guidance is required. Arts-based psychosocial practitioners, and particularly the leaders of organizations of non-profit and non-governmental organizations endeavoring to make community services more widely available in a safe and efficacious way are required to manage programs and activities that will support the communities that they serve. This planning process requires a multi-staged process including consultation, planning, implementation and evaluation. A logic model was chosen as a method to visually illustrate the core principles of the model across South African rural contexts, at its different stages from community engagement to implementation. Therefore the logic model has the potential to support psychosocial programs across a range of sectors and meets fund holders’ requirements for clear descriptions of operationalization, goals and outcomes.

### Background

Patel and De Beer (2021) found that South Africa’s most vulnerable farming communities continue to face challenges in receiving adequate mental health care because the extent of the problems is less visible due to poor data collection and care not being provided locally due to a shortage of community mental health professionals and poor access to public healthcare for rural communities.

This study focuses on the development of a logic model for a non-profit organization (NPO) working in the Western Cape of South Africa called sp(i)eel arts therapies collective [Sp(i)eel] in response to the lack of access to regulated psychosocial care within rural regions of South Africa. Sp(i)eel works with people in remote locations where it is known that there is limited access to health and social services as well as scarce financial resources, too few qualified and trained health professionals and stigma regarding mental health (Meyer, 2014; Swanepoel, 2020; Patel and De Beer, 2021). Programs take place in schools and community centers. The aims of their organization are to strengthen the family unit and the communities within which they live, through using creativity and imagination to activate a sense of ‘futurity’ requiring social change and healing from trauma (Barnes and Peters, 2002; Paulissen, 2009; Berman, 2014; Ngwenya et al., 2021). (Jones, 2005, p. 16) states that a culturally sensitive philosophy requires “…co-operation, collective responsibility and interdependence…”. sp(i)eel’s philosophy facilitates the imagining of futures beyond the present situation and applies the arts as a platform for the amplification of a community voice. An important feature of the sp(i)eel team is that the majority of practitioners are qualified as dramatherapists and applied theater practitioners. However, sp(i)eel has enabled practitioners to adapt their practice to become culturally sensitive through using all of the sensed media available to them. This means that in many contexts, practitioners use drawing, painting, singing, theater and, play underpinned by core concepts that relate to the vision of supporting the development of healthy communities. In other words, whilst drama and theater formed the greater premise for community engagement, new arts-based dialogs have developed through image, ritual and the non-verbal languages of the communities that they serve.

Sp(i)eel focuses on relationship-building with community leaders and organizations and these collaborations determine the longer term aims for the projects. Therefore, communities are respected for their unique strengths and challenges and the programs are adaptive to these. Whilst the majority of programs are being facilitated by arts practitioners and arts therapists, skill sharing is also a core part of the program whereby local community members (for example teachers and care workers) are trained to deliver or co-facilitate the programs. In this way, sp(i)eel aims to meet the demand for arts-based programs that are culturally sensitive and provides skills, knowledge and relational experience that can foster community development and posttraumatic growth (Peltzer, 1999; Reid, 2006; Holtman et al., 2011; Williamson et al., 2017; Artz et al., 2018). Like other NPOs working in this sector, sp(i)eel’s arts therapists have adapted their practice from a predominantly Eurocentric approach in which many South African Arts Therapists and psychosocial practitioners are historically trained, to focus on collaborative efforts between therapists, community artists and organizations that speak to the need for large-scale psychosocial support that is relevant to a South African context and cultural frame of reference (Oosthuizen et al., 2007; Berman, 2010; Goicoechea et al., 2014; Meyer, 2014; Fouche and Stevens, 2018). This study was conducted during the early stage implementation of sp(i)eel’s programs with children and their families in rural communities in South Africa.

### Mental Health in Rural South African Communities

There is increasing evidence that communities that have undergone socio-political and systemic traumas require a collective and co-produced approach to understanding and treating the ensuing problems that is sensitive to the language and cultural values of the indigenous population (Miller and Rasmussen, 2010; Ventevogel et al., 2011). Trauma in South African rural communities and transgenerational social disruption stems from a complex psychosocial history involving abuses of human rights, including unfair dismissals, unfair labor practices, assaults, rape and murder of agricultural workers or family members (The Black Association of the Agricultural Sector [BAWSI], 2016). Whilst extreme threatening measures for social control of farming labor is now illegal, it is only in recent years that the laws preventing remuneration of labor with alcohol (the *dop* system) has been actively enforced. This enforcement followed findings that the *dop* system had resulted in a transgenerational legacy of alcohol abuse linked to violence, crime, teenage pregnancies, fetal alcohol syndrome and domestic violence (London et al., 1998; London, 1999; The Black Association of the Agricultural Sector [BAWSI], 2016).

However, despite changes to the *dop* system, alcohol continues to be a major problem and plays an integral role in violent behavior, particularly toward women (Kiene et al., 2017; Hsiao et al., 2018; Manyema et al., 2018; Hatcher et al., 2019; Treves-Kagan et al., 2021). Swain et al. (2017) identified that South Africa’s mortality rate linked with interpersonal violence is seven times higher than the global rate. Peltzer (1999) linked violence exposure to the poor mental health of children within these communities. Out of a sample size of 149 children under the age of 17 years, it was found that 67% had witnessed violent events resulting in 8.4% of that population having PTSD symptoms. Swain et al. (2017) study on the prevalence of PTSD and PTSS with a sample of 320 adolescents from rural South African communities show a result of 6% meeting the criteria for PTSD and an additional 4% for PTSS. PTSD in rural communities is also associated with comorbid diagnoses of major depression, substance misuse disorder and anxiety disorder, the latter being particularly evident in young children (Swain et al., 2017). Adonis (2016) states that secondary trauma, poverty as well as a sense of powerlessness and helplessness perpetuate a cycle of trauma and limits socio-economic mobility (Adonis, 2016). Within this context, Sp(i)eel aimed to provide whole community support that began to build community resource, responsibility, autonomy and compassionate approaches to community care with the ultimate aim of developing a sustainable community resilience.

### Arts Based Psychosocial Practice

Arts-based psychosocial practice has been successfully delivered in a range of complex social contexts (see Boreham, 2004; Sliep et al., 2004; Dalrymple, 2006; Solomon, 2006; Sorsdahl et al., 2009; Jones, 2015; Langa et al., 2016; Zarobe and Bungay, 2017; Ebersöhn et al., 2018). However, even though Arts Therapists in South Africa are registered with the Health Professions Council of South Africa (HPCSA), unlike in the United States and many European countries (Waller, 1991), there are no qualified state registered Arts Therapies or Arts-Based Practitioner professionals available in public health. Sp(i)eel is required to operate outside of the usual health funded initiatives. In South Africa, the Arts Therapies are not offered as part of the regulated interventions (Prescribed Minimum Benefit), and most medical aid care plans do not cover community support, psychosocial practice or mental health support beyond those that are provided in mainstream care. Therefore, there are no standard operational and clinical guidelines for psychosocial practitioners working within complex socio-political contexts in South Africa because there has not been an explicit endorsement from government authorities the enabling integration of practice within existing operational structures.

The professional use of the arts in psychosocial programs refers to practice that applies a culturally sensitive and collaborative creative-expressive modality within a social context as the focus of their intervention and that this method works toward collective healing (Jordans et al., 2016). Arts-based practice is nuanced and highly varied and can include a variety of techniques and processes including image making, role-play, dance, movement, storytelling, music and improvisation (Johnson, 1982; Low, 2010; Jones, 2015; Shafir et al., 2020). Awadhalla and Qarooni (2018), refer to the core principles of psychosocial practice as being compassionate, non-judgmental and upholding the rights and beliefs of a community to support social cohesion and collaboration. Examples of the key issues identified by communities are, lack of social support, problems with marriage status, bereavement difficulties, work environment and trauma and social disruption (Upton, 2013). Effective practice can result in increasing community hope (Edwards et al., 2020), resilience (Ungar, 2017; Fouche and Stevens, 2018; Ebersöhn, 2019) social cohesion and infrastructure (Hansen, 2009; Awadhalla and Qarooni, 2018). Other benefits of community arts-based psychosocial practice are described as offering a safe space for expression (Berman, 2014; Colbert and Bent, 2018), applying the arts as a means to express difficult and painful feelings (Mueller et al., 2011; Goicoechea et al., 2014; Meyer, 2014; Colbert and Bent, 2018), building self-esteem (Cox et al., 2010; Goicoechea et al., 2014; Meyer, 2014; Colbert and Bent, 2018), developing positive relationships, social cohesion and a connection to community (Dos Santos and Pavlicevic, 2006; Goicoechea et al., 2014; Meyer, 2014; Colbert and Bent, 2018), reclaiming history and identity (Goicoechea et al., 2014; Meyer, 2014; Bodunrin, 2019; Clacherty, 2019) and promoting positive social change (Abad and Edwards, 2002; Gargarella, 2007; Berman, 2014; Meyer, 2014).

### The Use of Logic Models

The aim of the study being initiated by sp(i)eel was to visually delineate the relationship between existing practices, benefits and operations for their team to ensure parity of access, safe practice and reliable goals. This aim could be met in a range of ways, from an ethnographic approach to defining rich underlying patterns of relationships and dynamics that drive the organization or for example investigating the measurable impact of the organization’s activities and delineating practice according to the priorities and best practice available. No method is without its limitations and given the requirements to provide a road map that made the practices transparent within a reasonable timeframe, logic model development was seen as the best option. That said, the authors were aware that logic models can represent complex models of practice in overly simplified ways, that does not fully appreciate the organic interplay of voices and practices and the real-life complications that happen due to the unique nature of every group of people. This limitation was considered to be acceptable on the grounds that the process was the first stage of an iterative engagement with stake holders that would not be an end to itself but would provide a platform for further research developments. Logic models can be used to visually and systematically represent a complex program that has specific aims, benefits and desired outcomes (Afifi et al., 2011; Baxter et al., 2014; De-Regil et al., 2014). The logic model can be used to highlight specific dimensions to organize practice, with a summary of consensus of the impact. This can also include dimensions of activity that are required to achieve those outcomes. Essentially the logic model draws upon key concepts and arranges them in a linear diagram that illustrates a map of the program activities, with specific reference to the relationships between preparation, intervention and aims. Therefore, the logic model provides an effective tool for engaging key stakeholders, including funders, practitioners and participants. The logic model also provides a required level of detail of the program activity. Logic models have been used across a wide range of areas to this effect, providing a platform for consultation, dissemination and further research. A logic model is composed of the components of the program which are directly linked with the aims of that program and therefore can be evaluated independently of one another, providing the framework for comprehensive program evaluation and development as well as research planning, practice development and health intervention development (Baxter et al., 2014). We followed a process that identified the components of the logic model according to:

(1) Defining the problem.
(2) Describing the intervention (practice elements).
(3) Defining the perceived psychosocial benefits (immediate/intermediate and long term).
(4) Defining the goals.

## Materials and Methods

We utilized methods building upon the work conducted by the first author in health contexts (Havsteen-Franklin, 2014; Carr et al., 2021; Havsteen-Franklin et al., 2021). Ethical approval was obtained from Brunel University London and written informed consent was sought from all participants. The project was divided into 4 phases. Phase 1 used organizational policies and literature to map the goals of the program and operationalization. Phase 2 identified the perceived benefits. Phase 3 identified practitioner practice elements and phase 4 utilized a series of organizational focus groups to review the implementation of the logic model. As suggested by Carr et al. (2021) the process began by identifying the population who were being served and benefited from engaging in the program. We also critically assessed the ideological assumptions that underpinned the program. The findings were also explored by a wider practice group, and community members to verify findings to support ownership and implementation. To identify the operational elements, operational documents were reviewed. An overview of the literature was conducted to identify national and international models of practice, culturally sensitive approaches and research in relation to psychosocial practice with indigenous populations.

The first phase of the work resulted in the identification of the goals, perceived benefits and resources. Outputs are the direct result of the activities, the immediate results of a program, for example, the size of the communities and the frequency of delivery. Perceived benefits are changes that occur over time as a result of the activities and can be classified as immediate (changes occurring over 1–26 weeks), intermediate (occurring over 27–52 weeks), or long-term (taking 1–5 years to accomplish). The next phases of the logic model development involved the identification of interpersonal and community practice elements, perceived benefits and longer-term outcomes. The interpersonal and community practice elements are the arts-based activities and outputs identified with the program delivery.

Phases 1 – 3 resulted in a first draft of the program logic model. The draft was used to refer to in the team evaluation focus group discussions with 7 practitioners (see Table 1). In phases 2–3, the group developed the logic model further, using a focus group format to focus on the relationship of practice to immediate, intermediate and longer-term outcomes. In phase 4 the group members were invited to provide feedback on the developing logic model and to rank outcomes to be included in the logic model program design.

**TABLE 1:** Participant demographics.

Participant	Age	Education	Gender identity	Modality	Nationality	Years of experience
1	28	MA Dramatherapy, United Kingdom	Female	Dramatherapy	South African	5
2	24	Hons degree applied theater, SA	Female	Applied theater	South African	2
3	24	Hons applied theater, SA	Female	Applied theater	South African	2
4	39	MA art therapy, United Kingdom	Female	Art therapy	South African	9
5	33	Hons degree applied theater	Female	Applied theater	South African	7
6	23	Hons degree applied theater, SA	Female	Applied theater	South African	4
7	39	MA Dramatherapy	Female	Dramatherapy	South African	9

We explored the practice of the practitioners at interactional and operational levels and the perceived impact that these practices may have for the population.

### Phase 1: Defining the Problem

Sp(i)eel team held regular meeting to discuss, both in supervisory and management contexts best practice principles and the development of guidelines. The initial step for sp(i)eel was to consider what is currently being provided and to what benefit? How are arts-based practitioners conceptualizing the problem? Is this a social, family, cultural or health need? Are there specific populations who require more help than others? For example, does the problem relate to the agricultural workers, immigrants, women and children, the whole community or only those affected by social trauma? What is social trauma and is this particular to certain regions and if so why? Many communities that sp(i)eel worked with used a collective indigenous knowledge to support their understanding of social wellbeing and therefore sp(i)eel arts-based practice had evolved organically, responding to community needs that reframed and changed the parameters of a Western-centric approach (see Bedi, 2018). Practitioners identified an arts-based, decolonized, co-creation of knowledge about social trauma that was informed by and more readily accessible to the communities for whom the practice was intended (see Makanya, 2014). The next step of the project was to identify the longer-term goals for sp(i)eel providing psychosocial practice. The literature focusing on community arts-based psychosocial programs identifies resilience as a long-term goal underpinned by self-esteem and self-efficacy (Mueller et al., 2011). These findings were further verified using a focus group to assess the relationship between practice and outcomes. Further support of the long-term goals was provided by the systematic review conducted by Zarobe and Bungay (2017). Their resulting analysis of their findings indicated that participating in arts-based practice has an impact on intrinsic and extrinsic components of resilience. It should be noted, however, that mechanisms for arts-based practice were not described in their review.

In this context we are referring to collective resilience as the ability of groups and communities to activate a social capital system during and following social adversity. The program uses a theory identified by Ebersöhn (2017, 2020) called ‘flocking’. This describes a post-trauma growth response where people experiencing ongoing adversity are able to come closer to one another as a source of relational and emotional support. ‘When individuals use relationships as a way to access and mobilize resources, an enabling ecology is configured to foster positive adjustment’ (Ebersöhn, 2017, p. 29). This concept of collective resilience resonates with an indigenous African cultural system that usually prioritizes social values and collective identity over individualism. Collective resilience positions the community as an ecosystem where all people are affected by trauma within the social parameters regardless of symptomatology (Ungar et al., 2021).

The logic model was designed with the core principle of indigenous collective cultures in mind, which spanned collective resilience, child development, well-being, future learning and community post-trauma growth. The goals were ambitious, and the practitioners focused on the immediate fundamental building blocks to achieve relational and social goals. The longer-term goals of community resilience were considered in the context of a wider network of social support and practice where the arts played an integral role but were dependent upon other organizations such as community development NPOs, the Department of Community Safety, mobile health clinics and social workers from the Department of Social Development. It was noted that the creative processes of mapping and needs-based assessments can play a key role in developing and strengthening these networks.

### Defining Community Arts-Based Psychosocial Practice in Sp(i)eel

To investigate practitioner descriptions of arts-based psychosocial practice, the principal investigator (first author) proposed that there was a systematic inquiry into how practitioners describe their practice at an interactional level within the sessions and the associated perceived benefits of the arts-based interactions. A nominal group technique (NGT) was designed to gather data regarding consensus on perceived benefits and shared practice elements. The NGT has been widely used in health contexts to provide data about practice definitions and implementation (Van de Ven and Delbecq, 1972; Levine et al., 2006; Pastrana et al., 2010; Havsteen-Franklin, 2014; Rankin et al., 2016; Carr et al., 2021; Havsteen-Franklin et al., 2021). The method provides a structured exploratory forum and develops consensus about practice elements through thematic saturation (Saunders et al., 2018). The nominal group technique was selected from a range of possible focus group methods and chosen on the basis that the approach is time efficient and prioritizes a non-hierarchical approach to data collection. Essentially the four stages of the process are facilitated by a neutral actor, enabling both individual reflection and group exploration.

### Nominal Group Technique Stages

Stage 1. The questions are carefully identified by the whole group in relationship to the population, intervention, context and perceived benefits.

Stage 2. Individual responses are written down in silence within the group context.

Stage 3. All responses were shared and written up on a large board. Duplications were removed. The group explored patterns in the data until the data was organized into themes that form the essence of the groupings of statements. These themes were then discussed for their accuracy and meaning in the context of the question and adjusted according to the context and salience to practice.

Stage 4. The themes were ranked from 1 to 10. In this context the themes were ranked according to the relationship between practice and perceived benefit. 1 being a low salience and 10 being very high salience.

The investigator leading the project is a senior art psychotherapist working in the United Kingdom National Health Service and an experienced clinical-academic familiar with the use of the NGT. The investigator used a personal construct approach to guiding discussion within the NGT where questions focused on participant’s personal language and experiences. A personal construct psychology approach assumes that people develop personal constructs that guide behaviors as preferred polemic opposites. Therefore, personal construct psychology assumes that to understand the perceived benefits of arts-based psychosocial practice requires an understanding of underlying personal constructs (Winter, 2013).

Through synthesizing data that emerged in the collection of individual responses to the question, we observed patterns in the data that defined overarching themes. Participants organized the data into superordinate themes which allowed for shared understanding and agreement or disagreement with the theme (Fereday and Muir-Cochrane, 2008). The hypothesis being tested was whether participants had developed shared psychosocial models of practice and perceived benefits. Consensus was decided by discussion, using voting until there was unanimous agreement on language and the relationship between practice elements and perceived benefits. It was highly relevant that a common language about the use of arts was quickly established, where the single art therapist in dialog with applied theater practitioners and dramatherapists shared ways of practicing, particularly their stances and approaches to using the arts, sharing similar practice elements. This appeared to be relevant to and shaped by the communities that they were working with where historically arts had been used to bring communities together. Therefore, the clinicians employed by collect relevant data the investigator selected a purposive sample through recruiting arts-based practitioners employed by sp(i)eel (see Table 1).

The inclusion criteria for the participants (Table 1) followed the sp(i)eel employment guidelines in that the practitioners:

(1) Were qualified arts-based psychosocial practitioners (applied arts and arts therapies).
(2) Worked with communities that have experienced social adversity.
(3) Delivered arts-based psychosocial practice within a context local to the farming community (this may include schools and community centers).

### Phase 2: Perceived Benefits

Before the NGT began, development of the question resulted in the group defining the perceived benefits as observations made regarding the impact of the work commonly identified by the community members. We defined the question as “What do you see as the perceived benefits of your arts-based psychosocial practice for rural farming communities that have experienced the impact of significant trauma?” The participants agreed on five reliable criteria for each perceived benefit. The criteria were informed by psychosocial and evidence-based research methods (Elliott, 1989; Kazdin, 2017) and the participants agreed that the criteria for the constructs were that the perceived benefits were:

(1) Observed within 2-months after the delivery of the practice.
(2) Understood and agreed as being a perceived benefit by all members of the group.
(3) Perceived as a valid benefit to the population (i.e., related to the presenting issues and reasons the community members engaged in the practice).
(4) Reliably observed in practice.
(5) Influenced by arts-based practice.

### Phase 3: Practice Elements

A second NGT was facilitated to identify in-session practice elements that were perceived to impact on the perceived benefits. The practitioners were asked to describe practice elements that the practitioners feel confident in delivering and formed part of their practice. To determine the perceived relationship between practice and perceived benefit, the group agreed to respond to the question, ‘What Are the Practice Elements Used to Facilitate the Perceived Benefits?’

## Results

**FIGURE 1:**
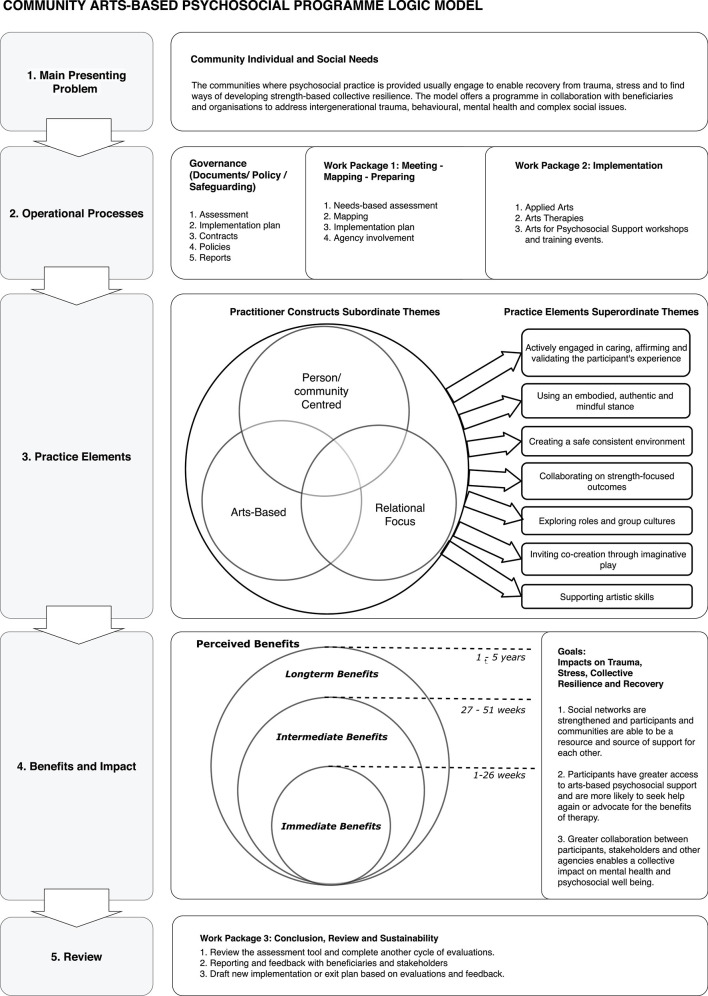
The community arts-based psychosocial program logic model. (1) Of the community arts-based psychosocial program logic model describes the presenting issues defined according to the community needs, available health data and literature. (2) Comprises the required documents, policies and work packages outlining the deliverables. During the first work package this is carefully documented adhering to organizational policies. The activities also include liaising with other organizations that can support the health and wellbeing of the community. Communities are engaged with sensitively, and self-referred to support a community psychosocial response to drug and alcohol abuse, teenage pregnancies, traumatic reactions to the working environment (e.g., PTSD) and where the there is significant child neglect. (3) Describes the practice elements identified through the focus group sessions which utilize arts for the purposes of community engagement to support a range of immediate, intermediate and long-term benefits. The approach used by the practitioners is primarily arts-based, person-centered with a relational focus. The community program may include a range of trust building exercises using drama, art, music and movement to provide a playful interactive program. The initial collection of statements identified supraordinate themes organized according to the categories of being (1) arts-based, (2) relational focus and (3) person centered practice (see Figure). Further thematic analysis produced 7 themes, (1) actively engaged in caring, affirming and validating the participant’s experience, (2) creating a safe consistent environment (3) using an embodied, authentic and mindful stance (4) collaborating on strength-focused outcomes (5) exploring roles and group cultures (6) inviting co-creation through imaginative play (7) supporting artistic skills (Figure 2). In Figure 1 (4) describes the goals and benefits as impacting on stress, resilience and recovery through building networks and a culture of acceptance and collaboration. In relation to perceived benefits, the focus group produced 12 interdependent immediate, intermediate and longterm benefits which were organized thematically into the following categories. The immediate benefits, were benefits that would be achieved during the program and included (1) establishing a stronger sense of self, (2) establishing a sense of a potential safe space, (3) regulating emotions (4) developing and sustaining healthy relationships. Intermediate benefits were (5) sustaining self-esteem, (6) validating and acknowledging other’s differences and diversity, (7) being reflective, (8) developing new narratives. Finally long-term benefits were identified as (9) being playful, (10) being creative, (11) creative learning (life skills) and (12) institutional learning. (5) Describes the steps required to review the program.

The results of practice elements and perceived benefits are described in Figure 1. The diagram in Figure 1 is divided into five parts which comprise the community arts-based psychosocial practice program.

### Immediate Perceived Benefits (Superordinate Themes)

#### Establishing a Stronger Sense of Self

A ‘sense of self’ referred to the experience of ‘me’ as reflective about self and others and growing to form a greater sense of agency and autonomy. Practitioners described a sense of self as being established in early infancy and through successful secure attachment patterns with parental figures. They also associated a sense of self with self-worth (self-esteem) and free will. The group described observations that poor early attachments, and/or significant stressors impacted on the development of a sense of self.

#### Establishing a Sense of a Potential Safe Space

Practitioners described this theme as potentiality and scope for developing conditions within which children, parents or families could exist without fear of severe harm or threat. Feeling safe can be compromised by erratic or disturbing behaviors, domestic violence or abuse, and significant stressors related to security of work or significant abuse. The ‘potentiality’ of safeness seemed important to the participants given the entrenched issues and the development of hope, acknowledging the long-term work of establishing safe emotional and physical conditions within which to live.

#### Regulating Emotions

Emotional regulation referred to the participant’s capacity to mediate and communicate feelings to another safely rather than expressing feelings in destructive or harmful ways, with reference to aggression and anger in close relationships. Practitioners said that aggressive behavior in families occurred in conditions of high vulnerability often when a sense of self and cultural identity was under threat.

### Developing and Sustaining Healthy Relationships

Practitioners described healthy relationships as dependent on a range of factors, which were also present in other themes. The group described this construct as the action of utilizing a range of interpersonal skills to make good interpersonal contact, be reflective about feelings and thoughts, being imaginative and playful with people and being contingently caring and compassionate, for example developing trust in a caring family context.

### Intermediate Perceived Benefits

#### Sustaining Self-Esteem

Participants’ successes and achievements can be impacted on by adverse events and further trauma. Practitioners closely associated self-esteem with self-worth and a self-ideal. It was evident that practitioners felt that the sustaining of self-esteem was necessary and resources, such as changing the culture of a community toward more supportive relationships was believed to support self-esteem beyond the direct impact of the practice.

#### Validating and Acknowledging Other’s Differences and Diversity

The practitioners described working with a community of between 200 and 900 people where the population speaks different languages and has heritages of different cultures. Practitioners described working closely with the emotional and relational dimensions of dispute which had often taken the appearance of entrenched belief systems and cultural divisions. They also described major stressors such as poverty, addictions and illnesses significantly impacting on perceptions of discrimination and vice versa.

#### Being Reflective

The practitioners felt that the development of a capacity to be contingently less reactive to interpersonal stressors and to be explorative rather than having fixed concrete ideas was a primary outcome of the work. They considered the action of being reflective as non-reactivity and therefore being more open and explorative.

#### Developing New Narratives

Practitioners felt that a strong benefit of the work, but secondary to many of the more relational, interaction focused outcomes, was the development of personal and collective stories that illustrate realistic potentials for seeing or experiencing themselves and their community in an open creative way rather than accepting fixed inherited narratives. Practitioners referred to restrictive transgenerational narratives about participants and their communities, their role, potential, identity, and relationships. New stories about self-potentials were an important part of the project, especially where the community traumas had not been acknowledged in historical records.

### Long-Term Benefits

#### Being Playful

Practitioners considered playing to be a way of being explorative, pretending and feeling free and safe to improvise and find new ways of using objects. Whilst they considered this a significant outcome that could be impacted upon, it was not considered to be frequently observed and the group felt there were fundamental factors as previously described that held higher importance for the population. The group described playfulness as part of an open and imaginative interaction with the world and other requiring a safe context.

#### Being Creative

Being creative was considered to be enabling personal innovative and/or unique ways of interacting or using arts forms that offered a new perspective about themselves, others and the environment. Whilst they perceived this as an important benefit, this was not felt to be as valued by the participants where building trusting and safe relationships was a primary concern.

#### Creative Learning (Life Skills)

The practitioners considered creative learning to be different to ‘being creative’ because there was an emphasis on imaginative cognitive reappraisal and use of creativity to affect changes to learning about ‘life skills’ and daily living. Whilst they perceived this benefit as being impacted on by the arts-based practice, it was not considered to be as established as many other benefits. For example, the practitioners spoke about creative learning as being about understanding and embedding new life skills through psycho-education, such as relationships, parenting and keeping safe.

#### Institutional Learning

Whilst participants considered institutional learning to be important to the community members, and an area that the practitioners impacted upon, this was considered to be the least important perceived benefit. The community as an institution having an infrastructure, policies and legislation was not clearly defined for the community members through engaging with psychosocial practice and therefore the theme of institutional learning was important to the community but was not considered to be one of the main perceived benefits.

## Phase 3: Practice Elements

**FIGURE 2:**
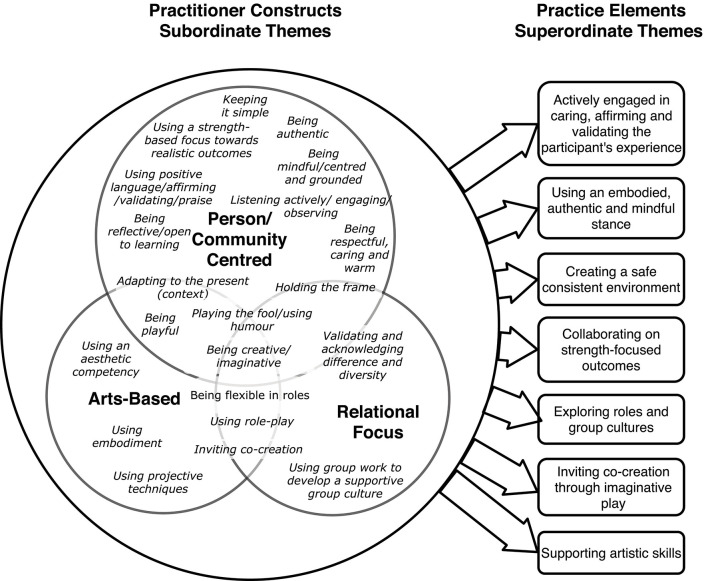
Practice elements themes.

The community arts-based psychosocial practice program utilizes a range of competencies that were identified and categorized into themes in a second NGT (Figure 2).

### Actively Engaged in Caring, Affirming, and Validating the Participant’s Experience

The practitioners stated that they want to be fully present in the sessions, they intend to be warm, caring and have a genuine interest in the participants’ wellbeing. They actively listened and were attentive to the participant’s non-verbal communications. The practitioners described adopting a stance of being mutually affected by the process, through empathy and emotional attunement.

### Creating a Safe Consistent Environment

The practitioners agreed that the safety and integrity of their practice relied not only on the sense of being reliably and continuously responsive and available, but also on the practical set-up of the space within which facilitation occurred. This included being on time and preparing for sessions, for example, making sure that the space could be used for the purposes of the group and that it was clean, quiet and non-threatening. Consistency included maintaining communication about the sessions, continuity of contact and that sessions were arranged in collaboration and in advance.

### Using an Embodied, Authentic, and Mindful Stance

This referred to an awareness of personal triggers and the intentional grounding of the practitioner through psychophysiological and interoceptive awareness, managing stressors enabling the practitioner to work from an open, relaxed and reflective stance to facilitate authentic relationship building. This in turn supports the practitioner’s ability to be flexible and respond to the needs of the group in the present moment, rather than rigidly following a session plan. Furthermore, arts practitioners emphasized the value of the use of the body and arts form to articulate emotions and non-verbal responses and cues.

### Collaborating on Strength-Focused Outcomes

This refers to the ability of arts-based practice to nurture creative responses to social contexts, challenging some of their assumptions, and building on their capacity to find creative solutions and ways of connecting with others. At the center of the practice creativity was seen as a personal resource and source of strength. The practitioners suggested that their psychosocial work often invites participants to collaborate in creative processes that elicit personal strengths such as determination, perseverance, the ability to form positive relationships, the capacity to develop aspirations, hopes and optimism, the discovery of a talent and enjoyment of an arts such as song writing. The emphasis is on a collaborative effort between practitioner and facilitator to discover these personal and group strengths.

### Exploring Roles and Group Cultures

Practitioners were aware of historical and social cultures in rural communities that were developed in relationship to ancestry, the farming environment, families, colonization, and de-colonization. This meant that practitioners helped participants to explore perceived roles that were based on their strengths, and others that limited opportunities and social contact. The practitioners stated that through the creative work they intended to grow a group culture built upon how the group defined healthy social values to impact on resilience and strengths.

### Inviting Co-creation Through Imaginative Play

The practitioners felt that an arts-based approach enabled a space within which new ideas, experiences and possibilities could occur within a playful environment. A core part of the process was allowing improvised play that led to an experience of co-creating and reimagining a sense of self, other, community and possible futures.

### Supporting Artistic Skills

Whilst it was deemed by practitioners as being very important to have artistic skills as a practitioner so that there was a sensitivity to the community arts esthetics, the practitioners said that they took a non-judgmental and supportive approach to artistic skill sharing. In other words, the work was more community and engagement focused rather than based on esthetic values.

## Phase 4: Reviewing the Logic Model

Following the NGT focus groups, the program logic model was implemented, reviewed and evaluated. To evaluate the implementation of the logic model, sp(i)eel organized a series of meetings with their arts-based practitioners to explore the challenges and successes of implementing the logic model and to identify lessons learned. Eight practitioners attended, five of whom were also involved in the NGT. Two were qualified Dramatherapists, two were Dramatherapy interns, and four were applied theater practitioners. Whilst other arts were not included, the group felt that the language and concepts described as part of the NGT supported an inclusive dialog. Key issues were raised and discussed relating to inclusivity of the population, post-colonial and cultural sensitivity and resources required. Sustainable funding is based on feedback and evaluation and therefore reflecting on the successes and challenges of implementing the logic model was imperative to the sustainability of the program. The following key themes were perceived as challenges to the implementation that required continuous attention. The logic model was then revised to address the suggested changes (Figure 1).

### Inclusive Use of Language

The practitioners wanted to be able to use a language to describe operationalization that did not draw rigid parameters around populations as could be understood by the term ‘community.’ For example, as a result of forced removals during the apartheid, many South Africans found themselves living in communities that they did not choose and therefore may not identify with. The practitioners discussed a helpful stance of recognizing that the program can create temporary communities drawing on key concepts of ‘flocking’ referencing location, interest, personal and social identities.

### Being Invited to Deliver the Program to Meet the Community Needs

There was also a theme concerning the vital role that the initial work package plays in setting up a program (Work package 1: Meeting, Mapping, and Preparing). The practitioners described an important challenge of being invited into spaces as opposed to entering a setting with a project uninvited which risks colonial re-enactments. It is also important to understand who invited the program and for what purposes. This challenge was mitigated through a detailed needs-based assessment.

Part of the implementation of this tool required a creative mapping exercise with both the stakeholders and beneficiaries that identified the participants’ risk and protective factors in their social context. In this way, the practitioners ensured that the development of the program spoke directly to the unique needs of the group.

### Establishing a Multi-Stakeholder Network

It became apparent that initial contact also required an extensive process of networking, whereby sp(i)eel made every effort to build relationships with individuals and organizations related to the program activity, to ensure sustainability of the program aims. This included health services, community safety officers, social workers, NGOs and potential funders. It was decided that where possible, the organization also investigated membership to existing collaborative structures within the local community.

### Ensuring Ethical Consent and Safety With the Community

Exploration concerning work package 1 also addressed the beginnings of co-creating a safe space for the work to take place. This process allowed transparency about what the program is about, why it is being delivered and to whom. This step mitigated the experience of ‘being done to’ and the fear that can come from not knowing the program’s aims or intentions. Early collaboration and transparency was considered to be essential to successful delivery of the program when all involved parties are informed. For example, the community practitioners that delivered a youth group on Fridays spent 2 days visiting the community members door-to-door and talking with the parents and the children to ensure they knew what the program was about and why their children were attending. At this stage it was noted that a key challenge was ensuring that parents recorded their consent in an ethical way. Further to this, finding safe spaces within the community was of importance where there are no clinics or safe public spaces apart from schools and community centers.

### Sustainability of the Benefits of the Program

The group explored Work Package 3 to include consideration of needs upstream, enabling the community to have further support and training. Participants can be actively invited to be part of the evaluation process to identify needs. The assessment may result in another round of programs, identifying other stakeholders such as community-led groups or sport activities or new initiatives such as the closed social media group that a group of learners created with the support of sp(i)eel where they can continue to communicate with each other beyond the program. This reduces the risk of programs being delivered with short-term benefits and no sustainable follow-up and ultimately supports social change. The practitioners also highlighted the need to sensitively and transparently report the evaluation so that it becomes a shared task of creating knowledge and learning between practitioners, participants and stakeholders. For example, the group felt that this should also include how the work and images are disseminated in reports and shared on social media.

### Participant’s Feedback

To gather initial feedback about the program logic model, an evaluation questionnaire was given to participants, living on farms across 3 sites who had participated in the program between 2019 and 2021. The questionnaire was designed to respond to the immediate and intermediate impacts of the project, particularly in relation to social systems and the use of arts to develop resilience as well as goals of enabling a safer environment and developing psychosocial wellbeing.

**FIGURE 3:**
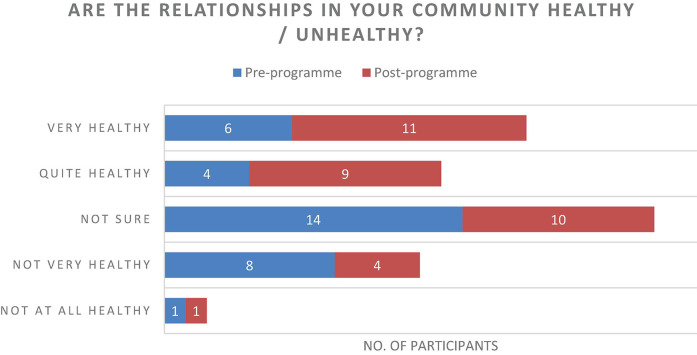
Are the relationships in your community healthy/unhealthy?

**FIGURE 4:**
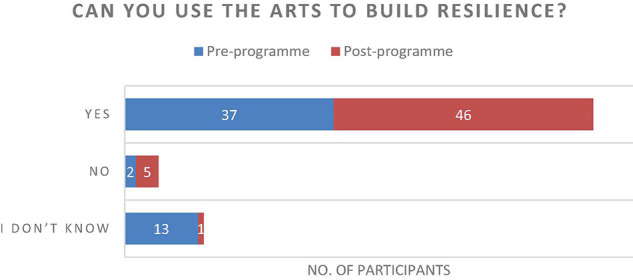
Can you use the arts to build resilience?

**FIGURE 5:**
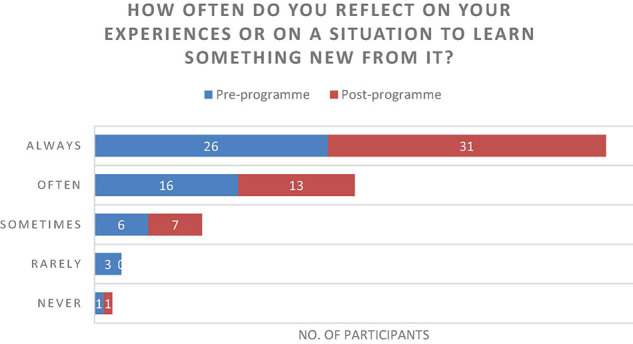
How often do you reflect on your experiences or on a situation to learn something new from it?

**FIGURE 6:**
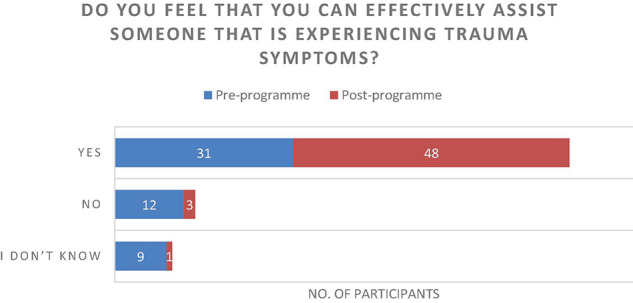
Do you feel that you can effectively assist someone that is experiencing trauma symptoms?

**FIGURE 7:**
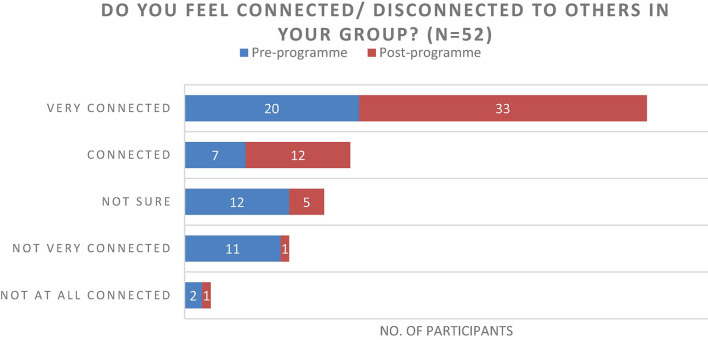
Do you feel connected/disconnected to others in your group?

The results of the questionnaire were positive and described an increase in individual’s experiences of connecting with one another (Figure 7), an increase of perceived healthy relationships (Figure 3) and a slight increase in the use of arts to develop resilience (Figure 4). Further to this, the data suggested that there was an increase in reflecting on experiences to learn (Figure 5), and a significant increase in feeling able to effectively assist someone with trauma symptoms (Figure 6). Together, this data points to participants strengthening social systems and developing relational resources to support their communities. One participant commented on the most important thing they have discovered, “People are very creative and they can sometimes use non-financial resources to assist others in trauma.” Another stated, “I have discovered creative ways to share information with others in my community.”

The use of arts was already familiar to the communities and therefore there was already a sense that arts made a contribution to resilient communities (*n* = 37). However, understanding and experiencing processes that draw on collaborative methods helped the communities to make sense of the significance of the relationship between resilience and arts, which was echoed in the data where respondents who initially answered ‘I don’t know’ (*n* = 13) decreased 9 (*n* = 5) whilst a positive response increased (from *n* = 37 to *n* = 46). Overall, the results suggested that the program model was having an impact that met the aims of the project.

## Discussion

This study describes a systematic approach to designing a program logic model and analyzing professional and participant feedback about the experience of the implementation of the model. Logic models are valuable tools for planning, monitoring, implementation and evaluating impact. The strengths of the study were that it demonstrated the feasibility of identifying and visually representing the operationalization of arts-based practices within complex social environments. The logic model was adjusted according to feedback and offers a valuable template to ensure guidelines are routinely employed to achieve the desired outcomes. However, there was often considerable debate about the nature of the research. Whilst the importance of providing timely systematic feedback to funders was understood by members, the value of making a systematic inquiry into practice that is often considered to be organic, responsive and collaborative was not as convincing. The concern underlying this debate was often about the problems of using Western medicalized models of psychosocial research that were incongruent with the ideas, beliefs and experiences of many of the people in the populations being served. One of the key concepts that drives sp(i)eel is that of flocking. This concept is pertinent given that the customs of the indigenous communities historically did not treat illness as a biomedical entity but as a personal and social experience. In alignment with a pluralistic constructed version of community health, the practitioners focus on the psychosocial collective experience of alcoholism, violence and the subsequent mental health conditions, rather than the biomedical model of symptom relief (Kleinman et al., 1978). Dialogs that begin with the community experiences are core to their practice. However, Sp(i)eel provides support for communities that provide laborers for a globalized fruit farming industry. In this sense, whilst the communities have an indigenous heritage, the dialog or pressure to conform with western commercially driven ideologies has produced uncertainties and a mix of cultural traits and identities that can no longer be identified as a homogamous cultural group (Hermans and Kempen, 1998). The practitioners are community led, responding to the social problems that have been identified by community members. However, many of the problems are caused by the problems encountered by becoming part of a global economy leaving the practitioners in a constant condition of uncertainty about their aspirations to have a longer-term impact on the social problems of those communities as the drive for production of the global community grows. Whilst the practitioners maintained an optimistic outlook, there was high sensitivity to colonization and the problems encountered even in forming a constructive dialog. The results demonstrated an experience-near subjective co-production through arts that was essentially about attempting to readdress a power imbalance, however, whilst practitioners were clear about longer term aspirations, there was also understandable hesitancy. Longer term benefits were perceived as being primarily about free will, enabling a sense of creative agency in the world that was supported by the wider institution where the institution learned from the community how to function and operate for the wellbeing of the community. In other words, the farming institution usually developed through generations of occupation on land claimed by colonial settlers, in many areas remains detached from an indigenous knowledge of collective wellbeing. With very few black indigenous researchers, the research project itself risked becoming a study conducted from a Eurocentric perspective about black people, mirroring early colonial records of problematized indigenous communities rather than embracing the positive qualities inherent within those societies (McEwan, 2003). It was apparent from the outset that the lead researcher was independently commissioned to offer impartial support with clarifying, facilitating and reflecting on social dialogs on the basis that sp(i)eel had made every effort to sensitively engage with indigenous languages for positive portrayals of identity, communication and community through their psychosocial arts-based work. In this way, the research attempted to make small steps toward greater participatory inclusion of all cultures. However, the project continued with the active acknowledgment that further research would be required offering a fuller exploration of the logic model elements with greater leadership and input from indigenous researchers and participants. One enabling factor for further research was that through community dialogs, the NPO was already experienced by agencies as a trusted resource and therefore the research as a collaboration was driven with the NPO’s aims in mind rather than as an external academic’s research interest. Whilst the iterative approach became a slow process to achieve results, this was necessary to meet the aims of the logic model and to be of value to the communities and stakeholders that were engaged with the process. One of the limitations determined by the process and data available was that we were unable to source evidence-based practice examples to support the operationalization of the arts-based psychosocial practice. We relied on qualitative studies, indigenous and cultural values as well as the experiences of the practitioners. Being guided by data that is close to the grass roots of the communities formed the basis of developing evidence from the community and NPO. This study adds evidence for the international community, given that to date the authors are not aware of any recorded descriptions of practice competencies and their resulting benefits visually contextualized within an operational framework.

## Further Research

This study demonstrated that there was a shared language and approach amongst practitioners, however, the authors would recommend further analysis of the relationship between practitioners, including practice skills, quality of interactions and theoretical underpinnings of their practices. For example process evaluation research where participant experience, reactions, mediators and unexpected pathways can be described in more detail (Moore et al., 2015). This is because the practitioners had a range of experiences, including trainings and understood themselves to be aligned with particular theoretical schools of thought and therefore participant feedback would support future developments of practice. This study built bridges between these schools of thought by treating each individual as embodying their personal theoretical perspective, rather than adhering to an external school of thought. Whilst arts-based practitioners appear to share common grounds of practice in other recent studies (Havsteen-Franklin et al., 2016, 2017; Carr et al., 2021), further research should investigate through a scoping study, how the practice is conducted and received and understandings of change processes with indigenous populations (Weiss et al., 2016). Another issue that arose during the study, is that other non-governmental agencies are often engaged in supporting rural communities with political, social and health concerns and further research regarding the optimum conditions to support change for communities with a history of major psychosocial disruption should be undertaken. Whilst the methods were systematically employed to provide full and open engagement of all professionals involved, it should also be noted that the sample size was small and the context was specific to a highly complex socio-economic cultural context where colonial and post-colonial values had impacted in many ways negatively on the indigenous population. Further research would need to be undertaken to see if the logic model is transferable to other complex social environments, for example in countries that have experienced extreme adversity due to natural or manmade causes. The authors also suggest that a feasibility study should first be conducted that is designed to be sensitive to the psychosocial environment, indigenous values and that uses measures that accurately reflect the desired change. For example, a stepped-wedge cluster randomized feasibility study (Hussey and Hughes, 2007; Liddy et al., 2015; Selby et al., 2019) could enable communities to be randomly allocated to a program whilst still receiving the program within reasonable timescales.

## Conclusion

Paulissen (2009) stated, “It is the way human beings imagine and engage their own futures. Without this dimension of futurity and imagination, we can hardly write a name we can call ours or articulate a voice we can recognize as our own.” This study developed a program logic model for a complex psychosocial project that aimed to enable young people, families and communities to engage with their own possible futures beyond the transgenerational post-colonial conditions. In this way the logic model was developed to describe the practice being delivered, the operationalization and the challenges faced to enable psychosocial change for a population living in a complex political and socio-economic environment. The key challenge was to create a model that provided a practice-based relationship to the context and addressed problems that were identified within rural communities. This inevitably required the model to be versatile whilst holding core practice elements in mind. The practice elements were defined according to the NGT process which produced specific themes capturing the interpersonal foci of the practice and the perceived benefits. To the author’s knowledge there has not been a comprehensive mapping of NPO delivery of psychosocial practice within a complex environment to date and as such this study can form the basis for further research of arts-based psychosocial practice with communities. Given that social and health resources are sparse in rural areas of South Africa, increasing access to psychosocial support and interventions can only be successful if existing resources are better researched and utilized. This study has the scope to support both increasing access to acceptable psychosocial interventions and helping to improve practice through identifying a model of operationalization described in the logic model.

## Data Availability Statement

The datasets presented in this study can be found in online repositories. The names of the repository/repositories and accession number(s) can be found below: https://brunel. figshare.com/articles/dataset/South_African_Arts-Based_Psycho social_Programme_Practice_Elements_xlsx/15036168.

## Ethics Statement

The studies involving human participants were reviewed and approved by Brunel University Research Ethics. The patients/participants provided their written informed consent to participate in this study.

## Author Contributions

DH-F coordinated the data collection, study design, and led the manuscript development process, carried-out the analysis of data and development, writing of the drafts and manuscripts, and oversaw the development and the implementation of the study. MS led the logic model implementation, coordination, and co-wrote the manuscript with the support of JJ and UC. All authors contributed to the article and approved the submitted version.

## Conflict of Interest

MS was the director of sp(i)eel at the time of writing this article. JJ and UC were commissioned to conduct arts-based psychosocial practice on behalf of sp(i)eel. The remaining author declares that the research was conducted in the absence of any commercial or financial relationships that could be construed as a potential conflict of interest.

## Publisher’s Note

All claims expressed in this article are solely those of the authors and do not necessarily represent those of their affiliated organizations, or those of the publisher, the editors and the reviewers. Any product that may be evaluated in this article, or claim that may be made by its manufacturer, is not guaranteed or endorsed by the publisher.

## References

[B1] Abad V. Edwards J. (2002). “Sing & Grow: a New Music Therapy Initiative to Meet Family Needs in Community Settings” in *Dialogue and Debate -Conference Proceedings of the 10th World Congress on Music Therapy.* (Eds) Fachner J. Aldridge D. (USA: World Federation for Music Therapy).

[B2] Adonis K. (2016). Exploring the Salience of Intergenerational Trauma among Children and Grandchildren of Victims of Apartheid-Era Gross Human Rights Violations. *Indo Pacif. J. Phenomenol.* 16 1–17. 10.1080/20797222.2016.1184838

[B3] Afifi R. A. Makhoul J. El Hajj T. Nakkash R. T. (2011). Developing a logic model for youth mental health: participatory research with a refugee community in Beirut. *Health Policy Plann.* 26 508–517. 10.1093/heapol/czr001 21278370 PMC3199040

[B4] Artz L. Ward C. L. Leoschut L. Kassanjee R. Burton P. (2018). ‘The Prevalence of Child Sexual Abuse in South Africa: the Optimus Study South Africa’. *S. Afr. Med. J.* 108 791–792. 10.7196/SAMJ.2018.v108i10.13533 30421700

[B5] Awadhalla M. S. Qarooni S. A. (2018). Disaster Response: psychosocial Support not Optional!. KnE Life Sci. 2018 14–25. 10.18502/kls.v4i6.3087

[B6] Barnes H. Peters D. (2002). Translating trauma: using arts therapies with survivors of violence. *S. Afr. Theatre J.* 16 157–183. 10.1080/10137548.2002.9687747

[B7] Baxter S. K. Blank L. Woods H. B. Payne N. Rimmer M. Goyder E. (2014). Using logic model methods in systematic review synthesis: describing complex pathways in referral management interventions. *BMC Med. Res. Methodol.* 14:62. 10.1186/1471-2288-14-62 24885751 PMC4028001

[B8] Bedi R. (2018). ‘Racial, Ethnic, Cultural, and National Disparities in Counseling and Psychotherapy Outcome Are Inevitable but Eliminating Global Mental Health Disparities With Indigenous Healing Is Not’. *Arch. Sci. Psychol.* 6 96–104. 10.1037/arc0000047

[B9] Berman H. (2010). Active Witnessing-Lefika la Phodiso’s Response to the South African Xenophobic Crisis. *J. Psycho Soc. Stud.* 4 1–31.

[B10] Berman K. (2014). “‘Imagination and Agency’ in Arts Activism, Education, and Therapies” in *Transforming Communities Across Africa.* ed. Barnes H. (United States: ProQuest Ebook Central).

[B11] Bodunrin I. A. (2019). Hip-hop and Decolonized Practices of Language Digitization among the Contemporary !Xun and Khwe Indigenous Youth of South Africa. *Critical Arts* 33 174–190. 10.1080/02560046.2019.1702070

[B12] Boreham N. (2004). A theory of collective competence: challenging the neo-liberal individualisation of performance at work. *Br. J. Educ. Stud.* 52 5–17. 10.1111/j.1467-8527.2004.00251.x

[B13] Carr C. Feldtkeller B. French J. Havsteen-Franklin D. Huet V. Karkou V. (2021). What makes us the same? What makes us different? Development of a shared model and manual of group therapy practice across art therapy, dance movement therapy and music therapy within community mental health care. *Arts Psychother.* 72:101747. 10.1016/j.aip.2020.101747

[B14] Clacherty G. (2019). The world in a suitcase: psychosocial support using artwork with refugee children in South Africa. *Participat. Learn. Action* 54 121–127.

[B15] Colbert T. Bent C. (2018). *Working Across Modalities in the Arts Therapies: Creative Collaborations.* London: Routledge. 10.4324/9781315559889

[B16] Cox S. M. Lafrenière D. Brett-MacLean P. Collie K. Cooley N. Dunbrack J. (2010). Tipping the iceberg? The state of arts and health in Canada. *Arts Health* 2 109–124. 10.1080/17533015.2010.481291

[B17] Dalrymple L. (2006). Has it made a difference? Understanding and measuring the impact of applied theatre with young people in the South African context. *Res. Drama Educ.* 11 201–218. 10.1080/13569780600671070

[B18] De-Regil L. M. Pena-Rosas J. P. Flores-Ayala R. del Socorro Jefferds M. E. (2014). Development and use of the generic WHO/CDC logic model for vitamin and mineral interventions in public health programmes. *Public Health Nutr.* 17 634–639. 10.1017/S1368980013000554 23507463 PMC4547471

[B19] Dos Santos A. Pavlicevic M. (2006). Music and HIV/AIDS orphans: narratives from Community Music Therapy. *Muziki* 3 1–13. 10.1080/18125980608538788

[B20] Ebersöhn L. (2017). A resilience, health and well-being lens for education and poverty. *S. Afr. J. Educ.* 37 1–9. 10.15700/saje.v37n1a1392

[B21] Ebersöhn L. (2019). *Flocking Together: an Indigenous Psychology Theory of Resilience in Southern Africa.* Switzerland: Springer. 10.1007/978-3-030-16435-5

[B22] Ebersöhn L. (2020). Collective resilience to global challenge: a collective wellbeing agenda to transform towards sustained equitable education. *Práxis Educ.* 15:e2016344. 10.5212/PraxEduc.v.16344.082

[B23] Ebersöhn L. Loots T. Mampane R. Omidire F. Malan-Van Rooyen M. Sefotho M. (2018). An indigenous psychology perspective on psychosocial support in S outhern A frica as collective, networking, and pragmatic support. *J. Community Appl. Soc. Psychol.* 28 332–347. 10.1002/casp.2371

[B24] Edwards B. M. Smart E. King G. Curran C. J. Kingsnorth S. (2020). Performance and visual arts-based programs for children with disabilities: a scoping review focusing on psychosocial outcomes. *Disabil. Rehabil.* 42 574–585. 10.1080/09638288.2018.1503734 30451026

[B25] Elliott R. (1989). “Comprehensive Process Analysis: understanding the change process in significant therapy events,” in *Entering the circle: hermeneutic Investigations in Psychology.* eds Packer M. J. Addison R. B. (New York: State Universtity of New York Press). 165–184.

[B26] Fancourt D. Finn S. (2019). “What is the evidence on the role of the arts in improving health and well-being?” *Health Evidence Network Synthesis Report.* (Italy: WHO Regional Office for Europe). 32091683

[B27] Fereday J. Muir-Cochrane E. (2008). Demonstrating rigor using thematic analysis: a hybrid approach of inductive and deductive coding and theme development. *Inte. J. Qual. Methods* 5 80–92. 10.1177/160940690600500107

[B28] Fouche S. Stevens M. (2018). Co-creating Spaces for Resilience to Flourish. *Voices World Forum Music Ther.* 18:2592. 10.15845/voices.v18i4.2592

[B29] Gargarella E. (2007). *Landmarks for Change: A Case Study Examining the Impact of a Community-Based Art Education Program on Adolescents*. Doctoral dissertation. Akron, OH: University of Akron.

[B30] Goicoechea J. Wagner K. Yahalom J. Medina T. (2014). Group Counseling for At-Risk African American Youth: a Collaboration Between Therapists and Artists. *J. Creat. Ment. Health* 9 69–82. 10.1080/15401383.2013.864961

[B31] Hansen P. (2009). *Psychosocial interventions: a handbook.* Switzerland: International Federation Reference Centre for Psychosocial Support

[B32] Hatcher A. M. Gibbs A. McBride R.-S. Rebombo D. Khumalo M. Christofides N. J. (2019). Gendered syndemic of intimate partner violence, alcohol misuse, and HIV risk among peri-urban, heterosexual men in South Africa. *Soc. Sci. Med.* 2019:112637. 10.1016/j.socscimed.2019.112637 31708236 PMC7296316

[B33] Havsteen-Franklin D. (2014). Consensus for using an arts-based response in art therapy. *Int. J. Art Ther.* 19 107–113. 10.1080/17454832.2014.968598

[B34] Havsteen-Franklin D. Jovanovic N. Reed N. Charles M. Lucas C. (2017). Developing a shared language within arts psychotherapies: a personal construct psychology approach to understanding clinical change. *Arts Psychother.* 55 103–110. 10.1016/j.aip.2017.05.002

[B35] Havsteen-Franklin D. Maratos A. Usiskin M. Heagney M. (2016). Examining arts psychotherapies practice elements: early findings from the Horizons Project. *Approaches Interdiscipl. J. Music Ther.* 8 50–62.

[B36] Havsteen-Franklin D. Oley M. Sellors S. J. Eagles D. (2021). Drawing on Dialogues in Arts-Based Dynamic Interpersonal Therapy (ADIT) for Complex Depression: a Complex Intervention Development Study Using the Medical Research Council (UK) Phased Guidance. *Front. Psychol.* 12:588661. 10.3389/fpsyg.2021.588661 33679511 PMC7930381

[B37] Hermans H. J. Kempen H. J. (1998). Moving cultures: the perilous problems of cultural dichotomies in a globalizing society. *Am. Psychol.* 53:1111. 10.1037/0003-066X.53.10.1111

[B38] Holtman Z. Shelmerdine S. London L. Flisher A. (2011). ‘Suicide in a Poor Rural Community in the Western Cape, South Africa: experiences of Five Suicide Attempters and Their Families’. *S. Afr. J. Psychol.* 41 300–309. 10.1177/008124631104100305

[B39] Hsiao C. Fry D. Ward C. L. Ganz G. Casey T. Zheng X. (2018). Violence against children in South Africa: the cost of inaction to society and the economy. *BMJ Global Health* 3:e000573. 10.1136/bmjgh-2017-000573 29515918 PMC5838395

[B40] Hussey M. A. Hughes J. P. (2007). Design and analysis of stepped wedge cluster randomized trials. *Contemp. Clin. Trials* 28 182–191. 10.1016/j.cct.2006.05.007 16829207

[B41] Johnson D. R. (1982). Principles and techniques of drama therapy. *Arts Psychother.* 9 83–90. 10.1016/0197-4556(82)90011-9

[B42] Jones P. (2005). *The Arts Therapies: A Revolution in Healthcare.* Hove: Brunner-Routledge.

[B43] Jones P. (2015). Drama For Life Keynote: trauma and Dramatherapy-play, space, language and relationships. *S. Afr. Theatre J.*

[B44] Jordans M. J. Pigott H. Tol W. A. (2016). Interventions for Children Affected by Armed Conflict: a Systematic Review of Mental Health and Psychosocial Support in Low- and Middle-Income Countries. *Curr. Psychiatry Rep.* 18:9. 10.1007/s11920-015-0648-z 26769198 PMC4713453

[B45] Kazdin A. E. (2017). Addressing the treatment gap: a key challenge for extending evidence-based psychosocial interventions. *Behav. Res. Ther.* 88 7–18. 10.1016/j.brat.2016.06.004 28110678

[B46] Kiene S. M. Lule H. Sileo K. M. Silmi K. P. Wanyenze R. K. (2017). Depression, alcohol use, and intimate partner violence among outpatients in rural Uganda: vulnerabilities for HIV, STIs and high risk sexual behavior. *BMC Infect. Dis.* 17:88. 10.1186/s12879-016-2162-2 28103834 PMC5248514

[B47] Kleinman A. Eisenberg L. Good B. (1978). Culture, illness, and care: clinical lessons from anthropologic and cross-cultural research. *Ann. Intern. Med.* 88 251–258. 10.7326/0003-4819-88-2-251 626456

[B48] Langa M. Masuku T. Bruce D. van der Merwe H. (2016). Facilitating or hindering social cohesion? The impact of the Community Work Programme in selected South African townships. *S. Afr. Crime Q.* 55 41–48. 10.17159/2413-3108/2016/v0n55a159

[B49] Levine D. A. Saag K. G. Casebeer L. L. Colon-Emeric C. Lyles K. W. Shewchuk R. M. (2006). Using a modified nominal group technique to elicit director of nursing input for an osteoporosis intervention. *J. Am. Med. Dir. Assoc.* 7 420–425. 10.1016/j.jamda.2006.05.004 16979085 PMC1839832

[B50] Liddy C. Hogg W. Singh J. Taljaard M. Russell G. Armstrong C. D. (2015). A real-world stepped wedge cluster randomized trial of practice facilitation to improve cardiovascular care. *Implement. Sci.* 10 1–11. 10.1186/s13012-015-0341-y 26510577 PMC4625868

[B51] London L. (1999). The ‘dop’ system, alcohol abuse and social control amongst farm workers in South Africa: a public health challenge. *Soc. Sci. Med.* 48 1407–1414. 10.1016/S0277-9536(98)00445-6 10369440

[B52] London L. Nell V. Thompson M. L. Meyers J. E. (1998). Health Status Among Farm Workers in the Western Cape - Collateral Evidence from a Study of Occupational Hazards. *S. Afr. Med. J.* 88 105–110. 9798496

[B53] Low K. (2010). Creating a space for the individual: different theatre and performance-based approaches to sexual health communication in South Africa. *J. Appl. Arts Health* 1 111–126. 10.1386/jaah.1.1.111/1

[B54] Makanya S. (2014). The missing links: a South African perspective on the theories of health in drama therapy. *Arts Psychother.* 41 302–306. 10.1016/j.aip.2014.04.007

[B55] Manyema M. Norris S. A. Said-Mohamed R. Tollman S. T. Twine R. Kahn K. (2018). The associations between interpersonal violence and psychological distress among rural and urban young women in South Africa. *Health Place* 51 97–106. 10.1016/j.healthplace.2018.03.003 29579700

[B56] McEwan C. (2003). Building a postcolonial archive? Gender, collective memory and citizenship in post-apartheid South Africa. *J. S. Afr. Stud.* 29 739–757. 10.1080/0305707032000095009

[B57] Meyer K. (2014). Making Fires: rethinking the possibilities of Creative Arts Therapy practice in South Africa. *J. Appl. Arts Health* 5 303–318. 10.1386/jaah.5.3.303_1

[B58] Miller K. E. Rasmussen A. (2010). War exposure, daily stressors, and mental health in conflict and post-conflict settings: bridging the divide between trauma-focused and psychosocial frameworks. *Soc. Sci. Med.* 70 7–16. 10.1016/j.socscimed.2009.09.029 19854552

[B59] Moore G. F. Audrey S. Barker M. Bond L. Bonell C. Hardeman W. (2015). Process evaluation of complex interventions: medical Research Council guidance. *BMJ* 350:h1258. 10.1136/bmj.h1258 25791983 PMC4366184

[B60] Mueller J. Alie C. Jonas B. Brown E. Sherr L. (2011). A quasi-experimental evaluation of a community-based art therapy intervention exploring the psychosocial health of children affected by HIV in South Africa. *Trop. Med. Int. Health* 16 57–66. 10.1111/j.1365-3156.2010.02682.x 21073640

[B61] Ngwenya N. Barnett T. Groenewald C. Seeley J. (2021). Complex trauma and its relation to hope and hopelessness among young people in KwaZulu-Natal, South Africa. *Vulnerab. Child. Youth Stud.* 16 166–177. 10.1080/17450128.2020.1865593

[B62] Oosthuizen H. Fouché S. Torrance K. (2007). Collaborative Work: negotiations between Music Therapists and Community Musicians in the Development of a South African Community Music Therapy Project. *Voices World Forum Music Ther.* 7:v7i3.546. 10.15845/voices.v7i3.546

[B63] Pastrana T. Radbruch L. Nauck F. Höver G. Fegg M. Pestinger M. (2010). Outcome indicators in palliative care—How to assess quality and success. Focus group and nominal group technique in Germany. *Support. Care Cancer* 18 859–868. 10.1007/s00520-009-0721-4 19701782 PMC3128732

[B64] Patel B. De Beer L. (2021). *Maverick Citizen Op-Ed: Mind Field: SA Urgently Needs a New Mental Health Policy. Daily Maverick.* Available online at: https://www.dailymaverick.co.za/article/2021-05-17-mind-field-sa-urgently-needs-a-new-mental-health-policy/ (accessed May 17, 2021).

[B65] Paulissen V. (2009). *African Contemporary Art: negotiating the Terms of Recognition.* Available online at: http://africultures.com/african-contemporary-art-negotiating-the-terms-of-recognition-9030/ [Accessed 4 February 2021],

[B66] Peltzer K. (1999). Posttraumatic stress symptoms in a population of rural children in South Africa. *Psychol. Rep.* 85 646–650. 10.2466/pr0.1999.85.2.646 10611795

[B67] Rankin N. M. McGregor D. Butow P. N. White K. Phillips J. L. Young J. M. (2016). Adapting the nominal group technique for priority setting of evidence-practice gaps in implementation science. *BMC Med. Res. Methodol.* 16:110. 10.1186/s12874-016-0210-7 27566679 PMC5002198

[B68] Reid S. J. (2006). Rural health and transformation in South Africa: opinion: sAMJ forum. *S. Afr. Med. J.* 96 676–677. 17019486

[B69] Saunders B. Sim J. Kingstone T. Baker S. Waterfield J. Bartlam B. (2018). Saturation in qualitative research: exploring its conceptualization and operationalization. *Qual. Quant.* 52 1893–1907. 10.1007/s11135-017-0574-8 29937585 PMC5993836

[B70] Selby N. M. Casula A. Lamming L. Stoves J. Samarasinghe Y. Lewington A. J. (2019). An organizational-level program of intervention for AKI: a pragmatic stepped wedge cluster randomized trial. *J. Am. Soc. Nephrol.* 30 505–515. 10.1681/ASN.2018090886 31058607 PMC6405151

[B71] Shafir T. Orkibi H. Baker F. A. Gussak D. Kaimal G. (2020). The state of the art in creative arts therapies. *Front. Psychol.* 11:68. 10.3389/fpsyg.2020.00068 32116898 PMC7012801

[B72] Sliep Y. Weingarten K. Gilbert A. (2004). Narrative Theatre as an Interactive Community Approach to Mobilizing Collective Action in Northern Uganda. *Fam. Syst. Health* 22:306. 10.1037/1091-7527.22.3.306

[B73] Solomon G. (2006). Development of art therapy in South Africa: dominant narratives and marginalized stories. *Can. Art Ther. Assoc. J.* 19 17–32. 10.1080/08322473.2006.11432281

[B74] Sorsdahl K. Stein D. J. Grimsrud A. Seedat S. Flisher A. J. Williams D. R. (2009). Traditional Healers in the Treatment of Common Mental Disorders in South Africa. *J. Nervous Ment. Dis.* 197 434–441. 10.1097/NMD.0b013e3181a61dbc 19525744 PMC3233225

[B75] Swain K. D. Pillay B. J. Kliewer W. (2017). Traumatic stress and psychological functioning in a South African adolescent community sample. *S. Afr. J. Psychiat.* 23 1–10. 10.4102/sajpsychiatry.v23i0.1008 30263182 PMC6138196

[B76] Swanepoel M. (2020). A collective breath: dreaming into a new model of mental healthcare. *Drama Ther. Rev.* 6 103–109. 10.1386/dtr_00052_1

[B77] The Black Association of the Agricultural Sector [BAWSI] (2016). *Farmworker Voices.* Available online at: http://www.bittergrapes.net/wp-content/uploads/2016/10/BAWSI-report.pdf [Accessed 4 February 2021]

[B78] Treves-Kagan S. El Ayadi A. M. Morris J. L. Graham L. M. Grignon J. S. Ntswane L. (2021). Sexual and physical violence in childhood is associated with adult intimate partner violence and nonpartner sexual violence in a representative sample of rural South African men and women. *J. Interpers. Viol.* 36 N7415–N7438. 10.1177/0886260519827661 30735091

[B79] Ungar M. (2017). Which Counts More: differential Impact of the Environment or Differential Susceptibility of the Individual? *Br. J. Soc. Work* 47 1279–1289. 10.1093/bjsw/bcw109

[B80] Ungar M. Theron L. Murphy K. Jefferies P. (2021). Researching Multisystemic Resilience: a Sample Methodology. *Front. Psychol.* 11:607994. 10.3389/fpsyg.2020.607994 33510683 PMC7835509

[B81] Upton D. (2013). “Psychological Factors and Health” in *Encyclopedia of Behavioral Medicine.* eds Gellman M. Turner J. R. (USA: Springer). 1556–1558. 10.1007/978-1-4419-1005-9_1562

[B82] Van de Ven A. H. Delbecq A. L. (1972). The nominal group as a research instrument for exploratory health studies. *Am. J. Public Health* 62 337–342. 10.2105/AJPH.62.3.337 5011164 PMC1530096

[B83] Ventevogel P. Ndayisaba H. Van de Put W. (2011). Psychosocial assistance and decentralized mental health care in post-conflict Burundi. 2000 – 2008. *Intervention* 9 315–331.

[B84] Waller D. (1991). *Becoming a Profession.* London: Routledge.

[B85] Weiss D. Lillefjell M. Magnus E. (2016). Facilitators for the development and implementation of health promoting policy and programs–a scoping review at the local community level. *BMC Public Health* 16:140. 10.1186/s12889-016-2811-9 26869177 PMC4751684

[B86] Williamson V. Butler I. Tomlinson M. Skeen S. Christie H. Stewart J. (2017). Caregiver Responses to Child Posttraumatic Distress: a Qualitative Study in a High-Risk Context in South Africa. *J. Traum. Stress* 30 482–490. 10.1002/jts.22215 29077999 PMC5698750

[B87] Winter D. (2013). *Personal Construct Psychology in Clinical Practice: theory, Research and Applications.* London: Routledge. 10.4324/9780203710371

[B88] Zarobe L. Bungay H. (2017). The role of arts activities in developing resilience and mental wellbeing in children and young people a rapid review of the literature. *Perspect. Public Health* 137 337–347. 10.1177/1757913917712283 28613107

